# Unexpected ward deaths: preventable?

**DOI:** 10.1186/2197-425X-3-S1-A66

**Published:** 2015-10-01

**Authors:** J Petit, B Malhomme, B Bihin, A Dive

**Affiliations:** CHU Dinant Godinne, Intensive Care, Yvoir, Belgium; Quality & Patient Safety Department, CHU Dinant Godinne, Yvoir, Belgium; Biostatistics, CHU Dinant Godinne, Yvoir, Belgium

## Introduction

We would assess the possible contribution of a future medical emergency team.

## Objectives

We analyzed the characteristics and circumstances of unexpected deaths that occurred in our center (450 beds) over the last ten years (2005-2014), with attention on risk factors that could have been present before the fatal issue.

## Methods

In our center, deaths are systematically reported to the « Quality & Patient Safety Department » via a questionnaire inquiring into the causes, circumstances and characteristics of death. Medical records of «unexpected » deaths (UD) (= “death that was not expected to occur at that time or/and under these circumstances”) were reviewed, after exclusion of patients with DNR orders. We analyzed the characteristics of this group, focusing on signs of clinical deterioration (written notes) within 48h before death (vital signs (VS): s. blood pressure < 90mmHg; heart rate: < 50 >130 BPM; SaO_2_ < 90%; polypnea), or on presence (1 or more) of the following presumed risk factors (PRF): confusion - contention - unable to call - inability to clear respiratory secretions independently. A comparison with non-unexpected deaths (NUD) has been made for systematically reported data (nursing and/or relatives presence; complications).

## Results

Out of 2188 ward deaths (ICU/ Emergency room/Operating room deaths excluded), 177 (8.1%) were considered UD (table 1). UD rate differed largely from one specialty to another (neurology 2%, orthopedic surgery 67%), with no significant difference between surgical and medical wards. UD occurred preferably in the early morning (06 - 08 am), irrespective of the week day. Most UD patients were admitted through ER (67%), stayed in the ICU (26%) or had surgery (36%) before. Presumed cause of death was respiratory (41.2%) or cardiac (27.1%).132 UD patients (75%) experienced either VS deterioration, or had 1 or more PRF (VS deterioration: n = 74; PRF: n= 18; both VS and PRF: n = 40). Presence of risk factors differed largely between wards; no risk factor (VS, PRF) was detected in most orthopedic surgery and internal medicine patients. PRF were more likely present in neurosurgery, rehabilitation and geriatrics.

**Table 1:** Table 1

2188 patients	UD (N = 177)	NUD (n = 2011)	P value	X²
Gender (male)	112 (63%)	1193 (59%)	ns	ns
Age (Moy/Med)	73/76	74/77	ns	ns
Lenght of stay (Moy/Med)	14/6	19/12		
Nursing presence	82 (46%)	740 (37%)	0,012	6,3
Relative presence	8 (4,5%)	1066 (53%)	p < 0,0001	153
Pulmonary embolism	12 (7%)	39 (2%)	p < 0,0001	16,7
Procedure complications	14 (8%)	27 (1,4%)	p < 0,0001	38,2
Cancer	32 (18%)	865 (43%)	p < 0,0001	41,8
Infectious complications	40 (22%)	687 (34%)	0,001	9,8

## Conclusions

There is a high incidence (75%) of VS deterioration and/or PRF presence in the period before unexpected hospital ward death. Presence and pertinence of each specific risk factor seems to depend on each specialty. This could contribute to identify patients who would benefit from early intervention.

